# Use of the Speckle tracking method for determining global parameters of heart contractility in healthy individuals

**DOI:** 10.1016/j.mex.2018.01.011

**Published:** 2018-02-02

**Authors:** V.E. Oleynikov, V.A. Galimskaya, S.N. Kupriyanova, N.V. Burko

**Affiliations:** Penza State University, Russia

**Keywords:** Correlation analysis of contractility indices, Global strain, Global strain rate, Normal range of strain, Echocardiography

## Abstract

The speckle tracking method allows one to quantify the temporal and spatial characteristics of myocardial contraction. Importantly, it does not depend on a scanning angle and allows one to record the movement of speckles in 2D mode in any direction, unlike tissue Doppler imaging. This examination is non-invasive, safe for patients, and economically more beneficial in comparison with other modern methods of assessing heart contractility: MRI and scintigraphy. Diagnostic thresholds are suggested for obtaining peak values of all types of global strains and strain rates by sampling a healthy group, which can reveal early signs of left ventricle contractility failure. Correlation relationships of deformation parameters between themselves and with left ventricular hemodynamic indices, as well as anthropometric parameters in healthy subjects highlight the features of heart contraction biomechanics. However, currently this method is scarcely studied because no generally accepted normal range of strain values exists.

• It is necessary to have sufficient qualification and skills in dealing with the XStrain™ Esaote software to obtain optimal values ​​of myocardial deformation.
• The study results expand the database of this software for users and determine the normal range of the left ventricular contractility parameters.
• The revealed interrelationships of strain values ​​in healthy individuals are relevant for understanding how the contractility mechanisms are altered in patients, and open up the prospect of studying the heart’s compensatory possibilities.

## Graphical abstract

**Figure:**
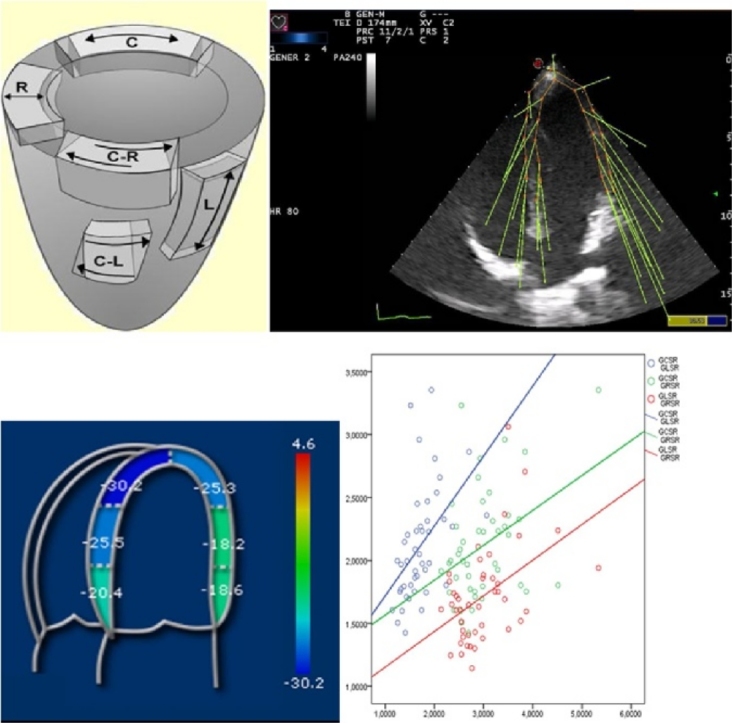

## Method details

Studying tissue deformation is a new diagnostic method in cardiology that makes it possible to quantify the regional contractility of the myocardium using two-dimensional echocardiography [1].

**Fig. 1:**
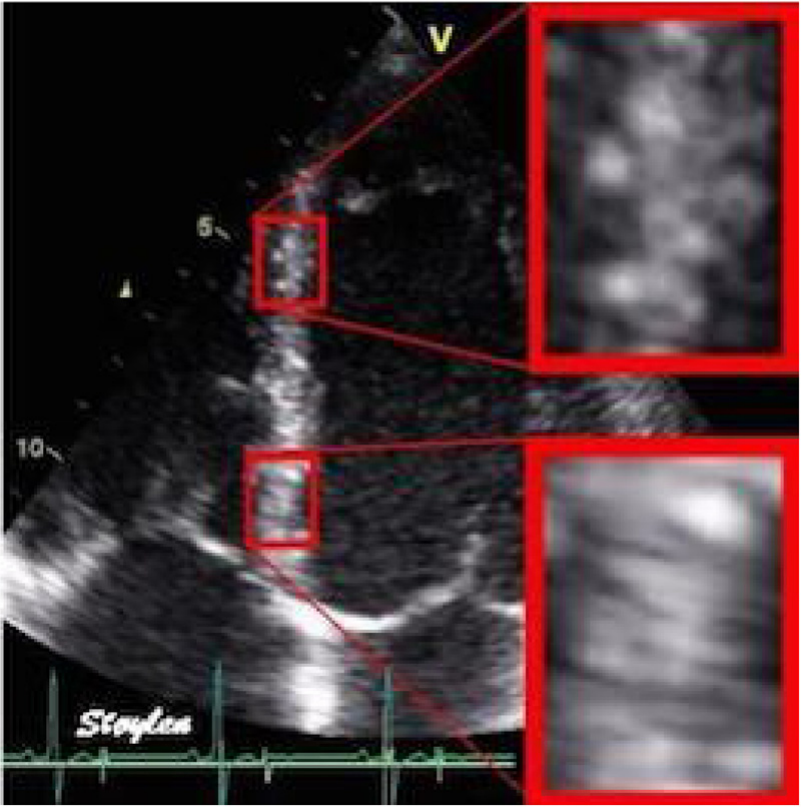
An example of gray scale spot imaging during inter-ventricular septum movement.

This technology allows one to record a unique pattern of gray scale spots, generated by ultrasound passing through myocardial tissues. Displacement of the gray scale spot (set) picture reflects the movement of the myocardium during systole and diastole, and displacement between the spots, respectively, the deformation of the myocardium (Fig. 1). This method is known as “speckle tracking”, which means “tracking of speckle points” [[1], [2], [3], [4], [5], [6], [7]].

**Fig. 2:**
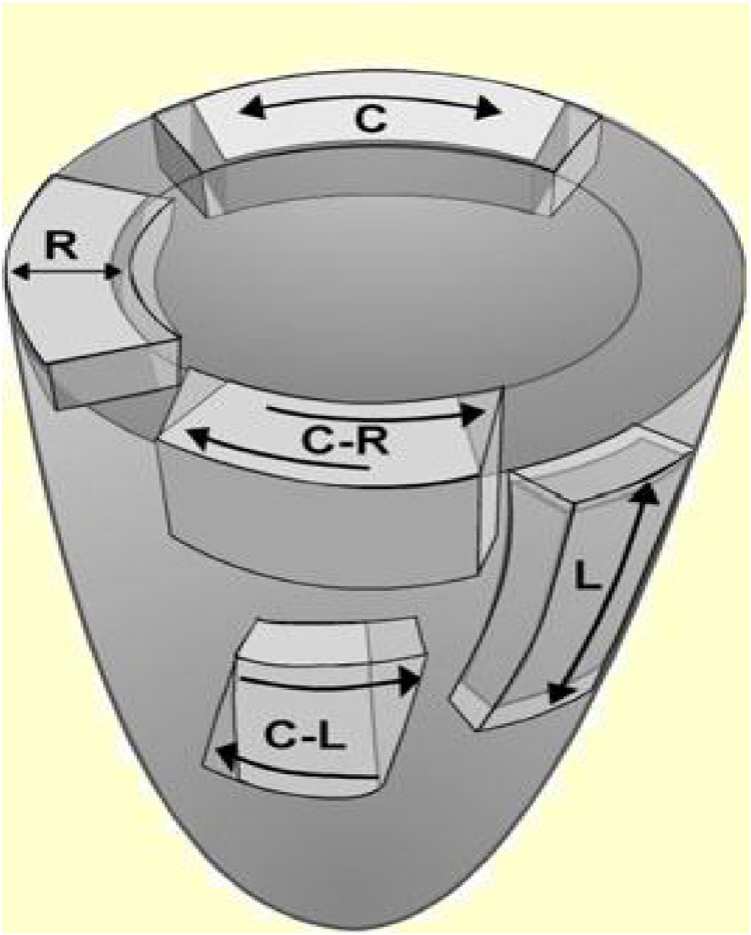
Directions of the left ventricular strain in three planes.

This technique has a number of advantages, in particular, its independence from a scanning angle, in contrast to tissue Doppler imaging [8]. Thus, it is possible to analyze myocardial strains and strain rates along three spatial axes, accordingly to the physiology of the heart muscle [[3], [5]]. Speckle-tracking echocardiography provides a picture of complex heart biomechanics by defining myocardial deformation in the longitudinal, circumferential, and radial directions (Fig. 2).

When using “speckle tracking”, no tethering effect exists because the strain is evaluated directly by the motion of gray scale spots of a certain area of the myocardium [4]. This technique is highly reproducible [[1], [9]] and does not require a high frame rate.

This study is non-invasive, safe for patients and economically more beneficial, compared with other modern methods of assessing heart contractility, namely, MRI and scintigraphy.

However, wide clinical application of this method is limited by the considerable variability of strain indices when using different software packages [1].

## Methods

A large-scale prospective study of 104 healthy volunteers (53 women and 51 men) was conducted (Table 1). Prospective criteria for recruitment included age >18 years, no history of cardiovascular or lung disease, no symptoms, the absence of cardiovascular risk factors (e.g., hypertension, smoking, diabetes, and dyslipidemia), no cardioactive or vasoactive treatment, and normal results on electrocardiography and physical examination. Exclusion criteria were athletic training, pregnancy, and body mass index >30 kg/m^2^. Blood pressure (BP) was measured in all participants immediately before the echocardiographic examination. Height and weight were measured using a calibrated stadiometer and scale, and body surface area was calculated according to the Dubois and Dubois formula. Body mass index was calculated by dividing the weight in kilograms by the height in meters squared (kg/m^2^). This study was approved by the local ethics committee of Penza State University, and written informed consent was obtained from all volunteers before they were screened for study eligibility.

**Table 1:** Baseline characteristics of the healthy subjects.

Variables	Value
Age, years	40 (26,5; 49)
Height, cm	175,2 ± 10,85
Weight, kg	77,7 ± 15,01
Body surface area, m^2^	1,9 ± 0,2
Body mass index, kg/m^2^	26,0 ± 4,17
Systolic blood pressure, mmHg	124 (120; 126)
Diastolic blood pressure, mmHg	80 (70; 80)
LV end-diastolic volume, mL	99,67 ± 26,83
Indexed LV end-diastolic volume, mL/m^2^	52,14 ± 11,82
LV end-systolic volume, mL	36,0 ± 13,7
Indexed LV end-systolic volume, mL/m^2^	18,75 ± 6,3
LV ejection fraction, %	63 (59,5; 69)

Echocardiography was carried out on the left side position with an ultrasonic scanner MyLabEsaote, a 2.5–5.5 MHz multidimensional sensor with a synchronized ECG from the extremities. The video clips were registered to the short-axis contours at the level of the mitral valve and papillary muscles, from the apical position of 5-, 4-, and 2-chamber images with respiratory arrest. Incorrect frames, such as intermediate positions between the long and short axes from parasternal access, as well as the absence of the apex or any wall of the LV were considered inadmissible. The computer analysis was based on the processing of digital signals of dynamic images of the heart with a frame rate in the range of 40–60 frames per second, using X-Strain software (Esaote, Italy), which allows one to estimate the longitudinal, circumferential, and radial myocardial strain and strain rate. The software algorithm tracks the frame-by-frame shift of the gray scale spots and extracts information about the strain and the strain rate of a specific myocardial segment in a given geometric position.

**Fig. 3:**
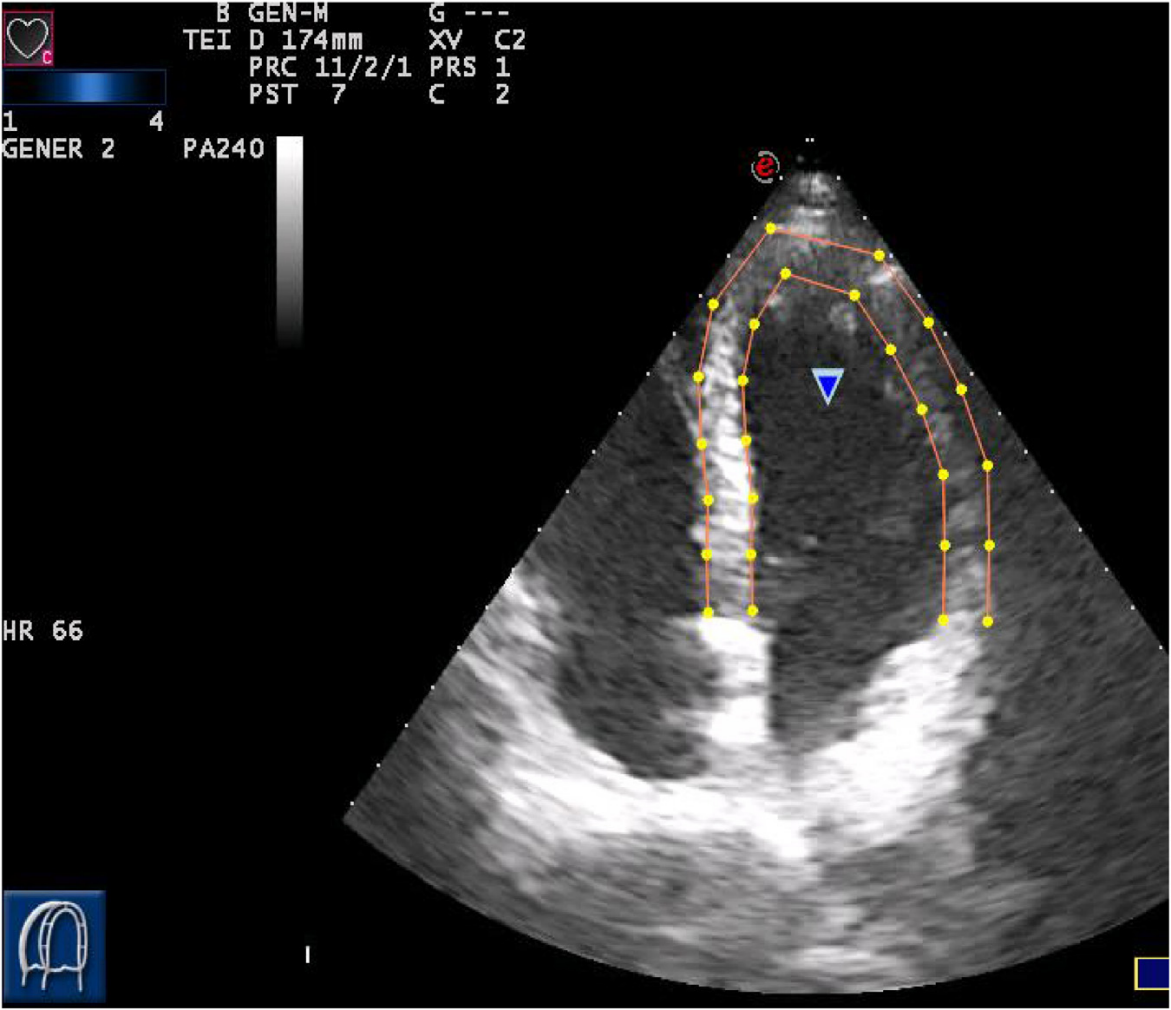
Tracing the endocardium and epicardium borders in the 4-chamber position.

For each patient, an initial image was selected at the end of diastole, which coincided with a QRS peak on ECG [1]. Good visualization of the intra-cardiac border is essential for accurate measurement. Patients having poor visualization of their echograms were excluded from the study. To improve the visualization of endocardium borders, we used the “gamma” option, which allows one to measure the gray scale intensity (Fig. 3).

**Fig. 4:**
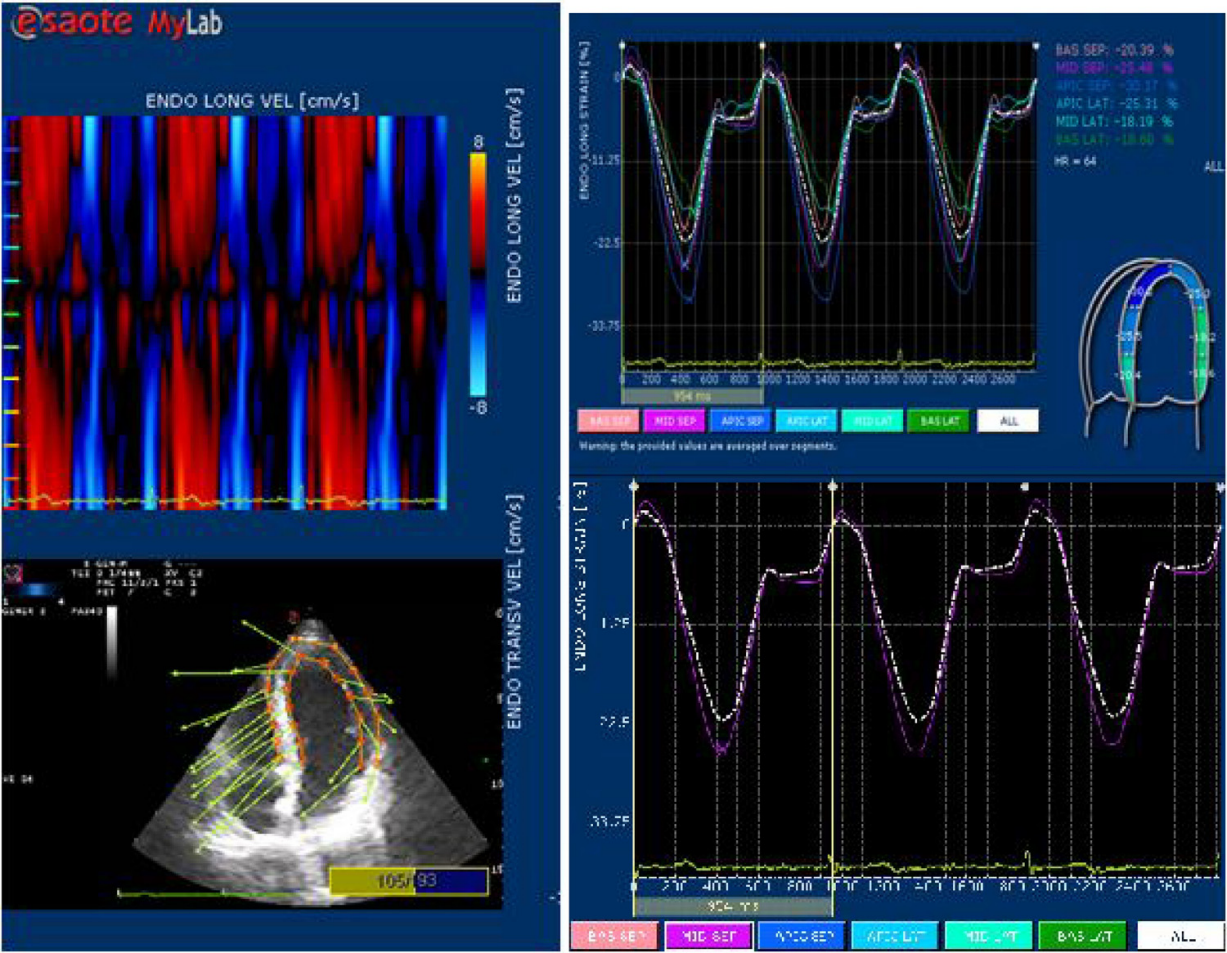
Four-chamber position. The endocardial and epicardial strain rate is shown as vectors. Graphs of all segments of the endocardial longitudinal strain. Longitudinal strain in the middle septal segment.

The anatomy of the analyzed segments should correspond to the LV scheme specified by the software, with the possibility of manual selection. The boundaries of the endocardium and epicardium were traced as a sequence of dots in the semi-automatic mode, with visual correction by the investigator. They should not be projected onto the papillary muscles and the aorta. Proper determination of endocardial borders is the most important condition for qualitative post-processing, requiring an examiner to have appropriate skills and sufficient experience in echocardiography. The parameters governing the myocardial deformation characteristics were evaluated in 16 segments. For each point, the strains and strain rates were automatically calculated, shown as vectors added to a two-dimensional image. Based on the dynamics of the cardiac cycle’s digital values, the software generates strain curves for each segment. We obtained strain values in the phase of interest in the cardiac cycle on the panel of these graphs. In addition, the strains and strain rate values for each segment were represented using color graphics (Fig. 4).

The global spatial and velocity indices were calculated as the arithmetic mean value of all segments [1]. The peak global endocardial values of the longitudinal, circumferential, and radial strain (in %) were determined, as well as the corresponding strain rates (sec-1) (Global Circumferential Strain/Strain Rate – GCS/SR), (Global Radial Strain/Strain Rate – GRS/SR). Longitudinal and circumferential strains have negative values in systole, and the radial one is characterized by positive values [3].

Regional deformation (strain) is a dimensionless value associated with changing the length of an object and is expressed as a percentage of its initial form. For one-dimensional objects, the deformation can be represented by elongation or shortening [[1], [3], [7]].

The rate of deformation (the strain rate) is the rate at which deformation occurs per unit time (c-1). The strain rate, as well as the definition of deformation allows one to estimate the degree of myocardial deformation [[1], [3], [7]].

From the echocardiographic parameters observed, the following were analyzed: the end-diastolic volume (EDV), the end-diastolic volume index (EDVI), the end systolic volume (ESV), the end systolic volume index (ESVI), the end diastolic LV dimension (EDD), the relative wall thickness (RWT), the stroke volume (SV), the LV ejection fraction (EF) by Simpson at the four chamber level, along the long axis of the left ventricle from the apical position, the left ventricular myocardial mass index (LVMI).

### Statistics

We constructed correlation models to determine the relationship between the contractile parameters of the left ventricle and its hemodynamic parameters, as well as anthropometric parameters, thus contributing to our understanding of heart biomechanics.

The correlation analysis methods with the Pearson coefficient (r) were used to determine the relationship between the variables. The distribution normality was established using the Kolmogorov-Smirnov test. The sensitivity of the selective Pearson coefficient for the corresponding index was determined as the probability of detecting connection tightness (1-β) in the general population.

## Results

Determining the descriptive characteristics of the left ventricular contractile function will contribute to refining the strain values, recorded on a MyLabEsaote scanner using XStrain™ Esaote software among healthy subjects. Table 1 presents the average peak global values of all strains and strain rate types, as well as factors reflecting the extent of their variability (Table 2).

**Table 2:** Summary of the characteristics of all global strain types.

		GLS	GLSR	GCS	GCSR	GRS	GRSR
Mean		21,00	1,50	25,18	1,84	35,62	2,52
Median		20,68	1,42	24,93	1,79	34,45	2,41
Standard deviation		2,73	0,35	4,02	0,42	8,50	0,62
Range		14,83	2,04	18,88	2,18	68,72	4,32
Minimum		15,60	1,02	18,21	1,18	18,06	1,02
Maximum		30,43	3,06	37,09	3,36	86,78	5,34
Percentiles	5	16,71	1,08	19,08	1,27	25,98	1,73
	10	17,75	1,13	19,68	1,36	27,70	1,96
	15	18,17	1,21	20,45	1,45	28,16	2,02
	20	18,67	1,25	21,79	1,48	29,56	2,10

Among all deformation characteristics, the largest value of the mean is the radial strain, followed by longitudinal and circumferential strains, which makes it possible to quantify the contribution of individual muscle types to the overall contractility. However, the radial strain index is characterized by high variability, which may limit its practical application. In contrast, owing to their small variability and low standard error, GLS and GCS can provide a more accurate estimate of the rate of contractility of the left ventricle. On this basis, we analyzed in detail the GLS score in healthy subjects in order to optimally determine the limits of its normal range. In addition, it has been established that a decrease in the global longitudinal deformation of LV is a sensitive marker of myocardial ischemia, which affects the cardiomyocytes’ contractile function at the subclinical stage of the disease [10].

The table also shows the values of all strains and strain rate types corresponding to the fifth, tenth, fifteenth, and twentieth percentiles. The mean values and standard deviations of the global deformation parameters obtained in this study can serve as a guideline for developing the normal range, taking into account the desired accuracy and reliability of the confidence intervals.

Only 5% of healthy individuals have GLS values below 16.71%; 10% below 17.75%; 15% below 18.71%, and 20% below 18.67%. The median value of 20.68% indicates that half of patients have GLS values that are both larger and smaller.

**Fig. 5:**
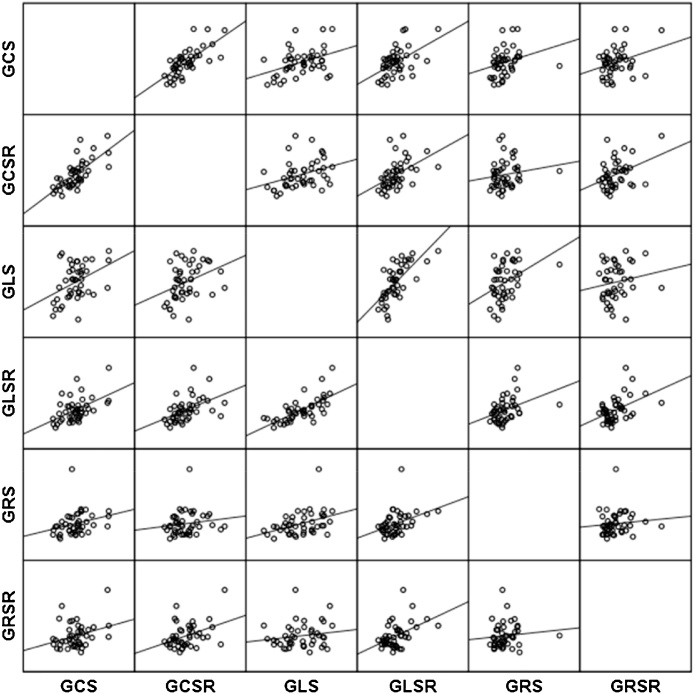
The matrix of scattering diagrams for pairwise correlation of strains and strain rates in healthy subjects.

It is recommended to define cut-off values to distinguish between healthy and sick individuals to determine the normal range, taking into account the optimal “sensitivity-specificity” ratio. This method, with high sensitivity, is useful for excluding the diagnosis, if the result is negative. However, a method with high specificity is useful for including the diagnosis, along with other possible factors if there is a positive result [11] (Fig. 5).

Table 3 presents the correlations of the spatial and velocity indices of contractility. The results are presented as a correlation matrix. Owing to diagonal symmetry, the lower part of the matrix is ​​not filled in, since it does not include additional information. One can see that all correlation coefficients are statistically significant at the level of 0.05; therefore, 95% confidence intervals are calculated for them.

**Table 3:** Pairwise correlation of the global peak strain and strain rate parameters in healthy individuals.

		GLS	GLSR	GCS	GCSR	GRS	GRSR
GLS	Pearson's Correlation	1	0,705	0,423	0,399	0,339	0,279
	Relevance		0,000	0,000	0,000	0,000	0,004
	95% confidence interval		0,613	0,280	0,253	0,187	0,122
			0,778	0,548	0,527	0,475	0,422

GLSR	Pearson's Correlation		1	0,475	0,562	0,353	0,567
	Relevance			0,000	0,000	0,000	0,000
	95% confidence interval			0,339	0,440	0,202	0,446
				0,592	0,664	0,488	0,668

GCS	Pearson's Correlation			1	0,750	0,246	0,338
	Relevance				0,000	0,012	0,000
	95% confidence interval				0,669	0,087	0,186
					0,813	0,393	0,474

GCSR	Pearson's Correlation				1	0,242	0,571
	Relevance					0,013	0,000
	95% confidence interval					0,083	0,450
						0,389	0,671

GRS	Pearson's Correlation					1	0,340
	Relevance						0,000
	95% confidence interval						0,188
							0,476

GRSR	Pearson's Correlation						1
	Relevance						
	95% confidence interval						

**Fig. 6:**
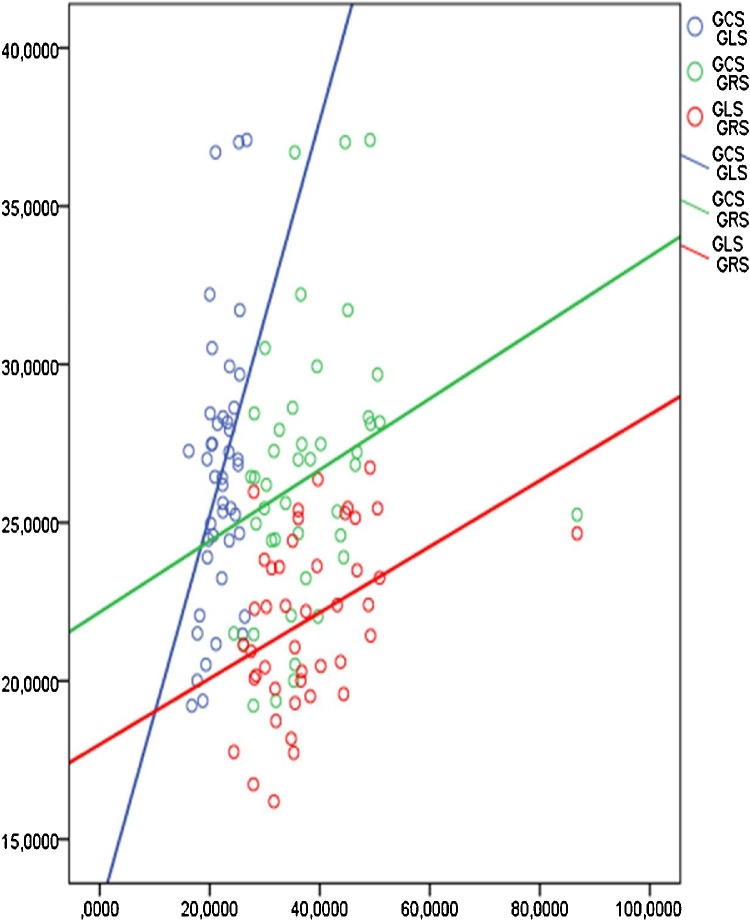
Graphs of the pairwise correlation of the myocardial deformation parameters in healthy individuals.

According to the table, the closest relationship is observed at pairwise comparison of GLS and GCS, with similar strain rate parameters, which is associated with a single structural-physiological mechanism underlying the magnitude and the time of contraction. In addition, high correlation coefficients are observed between GLS and GCS. This is due to the structural features of the two layers of the myocardium: the external and internal ones, respectively (Fig. 6).

**Fig. 7:**
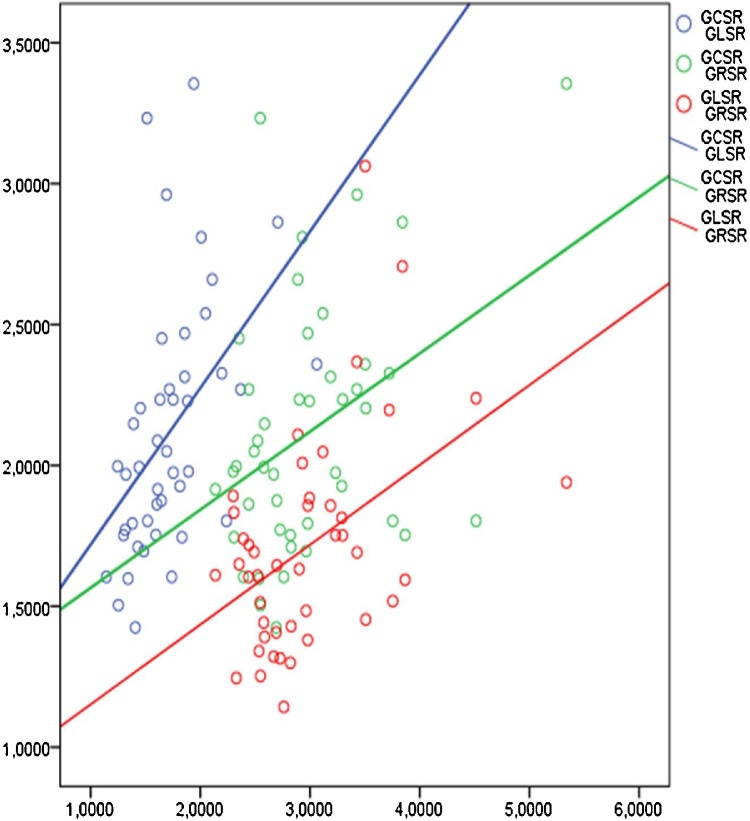
A pairwise correlation of the myocardial strain rates in healthy individuals.

The external oblique and the internal longitudinal muscles are represented by the same fibers, which start from the valve fibrous rings, go top-down, form a “vortex” at the apex of the heart and return to the fibrous rings already as a component of the inner longitudinal layer (Fig. 7).

The smallest correlation values of radial deformation with other types of strains and strain rates can be explained by the peculiarities of the distribution of the middle layer fibers, at right angles to the subepicardial and subendocardial layers and, consequently, the independence of these fibers’ contraction. Nevertheless, the reliable dependence of the majority of the revealed deformation characteristics indicates a well-coordinated cardiac contraction.

In analyzing the correlations presented in Table 4, note that statistically significant dependencies between the global characteristics of the longitudinal and circumferential strains with hemodynamic, structural heart parameters, as well as anthropometric parameters in healthy individuals are not shown.

**Table 4:** The pairwise correlation of all types of global peak strains and strain rates with hemodynamic, structural heart parameters and anthropometric parameters in healthy individuals.

		GCS	GCSR	GLS	GLSR	GRS	GRSR
BSA	Pearson's Correlation	−0,030	−0,102	0,043	0,163	0,077	0,342
	Relevance	0,849	0,510	0,783	0,292	0,620	0,023
	95% confidence interval						0,190
							0,478

Weight	Pearson's Correlation	−0,046	−0,077	0,006	0,173	0,084	0,353
	Relevance	0,767	0,617	0,968	0,261	0,587	0,019
	95% confidence interval						0,202
							0,488

LVPW	Pearson's Correlation	−0,005	0,022	−0,198	0,025	0,066	0,084
	Relevance	0,973	0,886	0,197	0,871	0,669	0,589
	95% confidence interval						

LVMMI	Pearson's Correlation	0,154	−0,004	−0,131	−0,082	0,377	0,097
	Relevance	0,319	0,981	0,398	0,597	0,012	0,532
	95% confidence interval					0,228	
						0,508	

BMI	Pearson's Correlation	0,011	0,023	0,033	0,195	0,152	0,321
	Relevance	0,942	0,885	0,831	0,205	0,326	0,034
	95% confidence interval						0,167
							0,460

EDVI	Pearson's Correlation	−0,096	−0,050	0,051	0,042	0,085	0,147
	Relevance	0,536	0,749	0,741	0,784	0,585	0,342
	95% confidence interval						

ESVI	Pearson's Correlation	−0,160	−0,122	−0,006	0,028	−0,066	0,109
	Relevance	0,301	0,432	0,967	0,857	0,669	0,480
	95% confidence interval						

IVS	Pearson's Correlation	0,139	0,129	−0,160	0,068	0,152	0,253
	Relevance	0,369	0,404	0,298	0,660	0,323	0,097
	95% confidence interval						

RWT	Pearson's Correlation	0,053	0,147	−0,177	0,114	−0,029	0,205
	Relevance	0,734	0,340	0,251	0,460	0,852	0,183
	95% confidence interval						

SV	Pearson's Correlation	−0,038	−0,031	0,039	0,076	0,149	0,256
	Relevance	0,807	0,843	0,803	0,623	0,335	0,093
	95% confidence interval						

EF	Pearson's Correlation	0,099	0,094	−0,002	−0,034	0,165	0,066
	Relevance	0,521	0,542	0,988	0,824	0,286	0,672
	95% confidence interval						

Note: BSA-body surface area, LVPW-left ventricle posterior wall; LVMI-left ventricle myocardial mass index; BMI-body mass index; EDVI-end-diastolic volume index; ESVI-end-systolic volume index; IVS-inter-ventricular septum; RWT-relative wall thickness; SV-stroke volume; EF-ejection fraction.

However, in the healthy population, there was a significant moderate direct relationship between GRS and LVMI. On the one hand, this dependence must be interpreted with caution, since GRS has the greatest variance. On the other hand, this regularity is justified, since the main thickness of the wall is the circumferential muscle, whose contraction causes radial deformation. In addition, the significant dependence of GRSR on the anthropometric characteristics (e.g., BSA, weight, and BMI) could be explained by the fact that a larger muscle size should contract in less time for effective cardiac output. The above-described interrelationships between the radial strain indicators and anthropometric data in healthy individuals are also reflected in other studies [12], which revealed higher values ​​for the radial strain in obese individuals (Table 5).

**Table 5:** Sensitivity of the selective Pearson correlation coefficient.

ρ	α	n	zα	z_1-β_	1–β
0,9	0,05	104	1,96	−12,76	0,999
0,8	0,05	104	1,96	−9,03	0,999
0,7	0,05	104	1,96	−6,71	0,999
0,6	0,05	104	1,96	−4,97	0,999
0,5	0,05	104	1,96	−3,53	0,997
0,4	0,05	104	1,96	−2,28	0,983

Note: ρ – the general correlation coefficient, α – the significance level, n – the sample size, zα – the critical value of the Fisher z-distribution, *z*_1−*β*_– the determining value of the Pearson coefficient sensitivity. 1-β – the sensitivity of the Pearson coefficient.

A correlation relationship (ρ), which exists within the entire population, with a probability of at least 98%, was detected for a sample size of n = 104.

## Discussion

In order to obtain the optimal values of left ventricular myocardial deformations by a MyLab90 Esaote ultrasound scanner, one must know how to work effectively with the XStrain ™ Esaote software and how to monitor all obtained deformations, with the possibility of manual correction at all stages of information retrieval offered by this software package. Therefore, we have introduced specific practical guidelines intended for specialists.

The correlations of the strain indices in healthy individuals, revealed in this study, have not been found in any available professional literature. The correlation patterns of deformation parameters were obtained from a small group of patients, which is a limitation of this study. Therefore, the relative reliability and high sensitivity of the obtained data, along with the correlation sensitivity coefficient, were used for optimal interpretation and interpolation of these results for the general population.

The correlation relationships of certain types of strains and the strain rates between them highlight the features of cardiac contraction biomechanics, which is relevant for understanding how the contractility mechanisms are altered in patients. It also opens up the prospect of studying the heart’s compensatory possibilities with optimal therapeutic and diagnostic correction. The other reliable correlations that were revealed, in particular, the signficance of the radial strain parameters in healthy individuals, indicate the dependence of cardiomyocyte contraction on body weight, which can predict the expended myocardial resources and the extent of their compensatory possibilities.

Determining the normal range of various physiological factors is a difficult task when a new technique is introduced into practical medicine. The problem of choosing the threshold values ​​of contractility by the speckle tracking method has been solved in here only in part, since its sensitivity can be determined by examining the population of patients with confirmation of the left ventricular contractility disturbance by other investigative methods. Therefore, we are planning a large-scale study on deformation characteristics in patients with CHD using the results obtained in this study.

All conclusions are mathematically justified, the application conditions and the results of the correlation analysis were verified, and exploratory regression analysis was performed.

## Conclusions

When using the new “speckle tracking” method it is necessary to acquire reliable data on various parameters of cardiac deformation. This will enable one to establish an algorithm for obtaining the specific deformation values needed to reveal heart biomechanics regularities in healthy subjects, in order to determine the normal values of these parameters for subsequent use in the diagnosis of various heart diseases.
